# Occult fractures in low-energy trauma—A retrospective forensic case series

**DOI:** 10.1007/s10140-026-02462-6

**Published:** 2026-03-30

**Authors:** Giuseppe Basile, Vittorio Bolcato, Luca Bianco Prevot, Michela Basile, Lucio Di Mauro, Livio Pietro Tronconi

**Affiliations:** 1 Department of Biomedical Sciences and Public Health, University “Politecnica Delle Marche” of Ancona, 60124 Ancona, Italy; 2 Research Center of Legal Medicine and Risk Management, Maria Cecilia Hospital, GVM Care & Research, 48033 Cotignola, Italy; 3 Astolfi Associati Law Firm, Milan, Italy; 4 Maria Beatrice Hospital, GVM Care and Research, 50121 Florence, Italy; 5 Department of Biomedical and Dental Sciences and Morpho-Functional Imaging, University of Messina, 98122 Messina, Italy; 6 Legal Medicine, Department of Medical, Surgical and Advanced Technologies, University of Catania, 95123 Catania, Italy; 7 Department of Health and Life Sciences, European University of Rome, 00163 Rome, Italy

**Keywords:** Occult fractures, Delayed diagnosed injuries; Low-energy trauma, Medical malpractice, Emergency medicine, Forensic traumatology, Musculo-skeletal imaging, Forensic medicine

## Abstract

**Background:**

Occult fractures (OFs) are inapparent fractures on initial plain radiographs, retrospectively diagnosed through second level imaging. The reasons for further diagnostic investigation may be the persistence of symptoms, or various personal needs, including insurance related. OFs, typically associated with low-energy trauma, are often underdiagnosed in the acute phase; however, they are relevant in the medico-legal field, as failure to identify them or delays in diagnosis and treatment may determine disability and give rise to complaint. Authors aim to describe OFs observed in a forensic case series.

**Methods:**

A retrospective study was conducted on technical expert consultations carried out between 2018 and 2024. Patients who sustained low-energy trauma with no evidence of fracture on plain radiography, followed by a later diagnosis of OF, were enrolled. Informed consent was signed. In each case, demographic data, trauma characteristics, diagnostic timelines, fracture locations, and details of any compensation claims were collected.

**Results:**

312 cases of OFs were included, with a prevalence at the sacrum (29.5%), posterior pelvic ring (18.8%), carpal scaphoid (16.0%). In 68.5% of cases, the diagnostic delay exceeded 15 days. More than two thirds of the anatomical sites involved by OFs typically require conservative therapeutical management. In 41.3% of occult fracture cases, a claim for alleged malpractice compensation was filed, in 23.7% claiming an increased damage due to diagnostic–therapeutic delay. Failure to recommend clinical–radiological follow-up, as well as inadequate documentation of signs and symptoms and of trauma dynamics, were also found.

**Conclusions:**

Occult fractures represent a critical issue in medico-legal practice as the increasing time from trauma to diagnosis may lead to litigation. The adoption of diagnostic protocols for low-energy trauma in selected settings that account for the risk of occult fractures, together with enhanced clinical assessment and documentation, is essential to prevent complaints.

**Graphical abstract:**

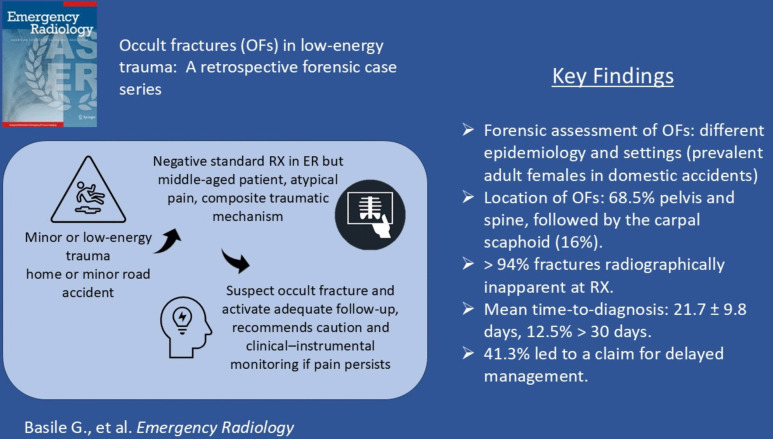

## Introduction

Musculoskeletal injuries are one of the most common reasons for accessing healthcare services following trauma, even when the traumatic event is mild in intensity and minor in dynamic [1]. In low-energy trauma, some injuries may more often escape initial diagnosis as the clinical presentation is often subtle, and trauma dominated by distortion-type mechanisms [2–4]. They often occur in the domestic setting, road traffic accidents, or non-agonistic sport-related injury. An “occult fracture” or “radiographically negative fracture” refers to a bone injury that is not initially visible on standard radiographic projections, either due to minimal displacement or technical limitations of the imaging modality. Follow-up radiographs, or more often second-level imaging techniques such as CT or MRI, later reveal the fracture, thus influencing clinico-therapeutic path [5]. This complex category of injuries belonging to delayed diagnosed injuries (DDI) is termed occult fracture even when confirmed by second-level imaging or retrospectively identified, prompted by persistent or recurrent symptoms [6].

Such fractures are recognized only during clinical follow-ups, weeks or months after the initial event, when persistent pain, functional limitation, or disability need further diagnostic investigation [7]. There is also a frequent recourse to forensic assessments for insurance indemnity. This may stand for a further reason to pursue in-depth investigation and confirmation of occult fractures.

Additional diagnostic challenges include age of the patient, both in terms of growing individuals and in the elderly, as certain anatomical sites are more prone to missed diagnoses (e.g., elbow, ankle, wrist, scaphoid, and foot), as are deep fracture or those confined to trabecular bone, without cortical involvement. These cases require careful interpretation based on the trauma dynamics, clinical symptoms, and radiological signs suggestive of acute injury, even when these are subtle or absent [3, 8].

Thus, in the forensic context, it is not solely the magnitude or mechanism of the trauma that determines the clinical and legal implications of musculoskeletal injury, but rather the timeliness and accuracy of the diagnosis, as well as the appropriateness of the clinical and rehabilitative management [9]. These aspects are crucial for achieving full clinical recovery, work ability, and thus return to normal daily, sporting, and recreational activities. Failure to detect or delays in diagnosing these fractures can lead to clinical complications, prolong rehabilitation, and increase disability. In recent years, there has been an increasing number of legal disputes related to delayed or missed diagnoses of these minor fractures, or to inadequate communication, management, or follow-up within the clinical and rehabilitative process [10–12]. Even apparently minor injuries can have significant long-term functional consequences, affecting work, leisure, and overall quality of life [13, 14]. These circumstances highlight the need for closer integration between clinical, radiological, and medico-legal ability to improve diagnostic pathways and reduce diagnostic errors or omissions, particularly in emergency and urgent care settings.

The present study aims to retrospectively analyze a forensic case series of low-energy trauma cases with later diagnosis of occult fracture, to characterize their clinical and medicolegal features. The goal is to identify areas for improvement both clinical and medicolegal in the management of patients presenting to emergency departments after low-to-moderate energy trauma.

## Patients and methods

### Study design

The study was designed as a retrospective observational analysis based on a series of medicolegal consultations conducted between 2018 and 2024. The cases were derived from both court-appointed and party-appointed technical evaluations, within the indemnity and/or compensation domain. The data were collected anonymously, recorded in a dataset for analysis, and shown in aggregate. As an observational study involving volunteer participants, ethic committee review was waived. All subjects, at consultation, gave informed consent to data processing for scientific research purposes. The analysis focuses on the most frequently involved anatomical sites, diagnostic timing, frequency of compensation claims, and factors associated with litigation.

### Inclusion and exclusion criteria

The definition of occult fractures was based on the concurrent presence of the following three criteria:

- Low-energy trauma, defined as a fall from a height not exceeding one meter, or a road traffic collision at a speed below 30 km/h (when documented), or described in the medical record as “minor” or “low-energy”.
- Initial radiographic assessment negative for fracture (plain X-rays interpreted as normal).
- Later diagnosis of fracture confirmed through advanced imaging (MRI or CT), performed due to persistent clinical suspicion or for medicolegal purposes.

Cases were included if they met the following conditions:

- Patients aged 18 years or older.
- Complete documentation available for review and analysis.

The following were excluded:

- Patients with high-energy trauma or polytrauma defined as major road traffic accident dynamics (e.g., vehicle rollover, ejection from a motorcycle), multiple fractures or involvement of multiple body regions requiring priority diagnostic assessment and treatment (such as fracture-related head trauma and/or concussion, major fractures requiring referral to a trauma center), described as “major trauma” or “high-energy trauma” within Health Records.
- Pathological fractures of neoplastic or metabolic origin.
- Incomplete or insufficient medical documentation.
- Pediatric patients (age < 18 years), due to the distinct anatomical and physiological characteristics of the growing skeleton.

### Data collection

For each case, the following data were collected: demographic information, trauma dynamics and contextual details of the event, anatomical location and type of occult fracture, time interval between trauma and definitive diagnosis of occult fracture (timetodiagnosis, TTD), details of any compensation claims filed, including medicolegal grounds for the request. The variables are specified in Table 1.

**Table 1:** Variables collected for each patient

Category	Variable
Demographic data	Age, sex
Trauma	Types of events (fall, collision, etc.)
Fracture site	Anatomical location
Initial imaging	Result of conventional radiography
Advanced imaging	Type (MRI or CT), findings
TTD (TimetoDiagnosis)	Interval between trauma and confirmed diagnosis (in days)
Medicolegal outcome	Litigation initiation and motivation

### Data analysis

Data was analyzed using both descriptive and statistical methods. Absolute and percentage frequencies were calculated for categorical variables, while means and standard deviations were computed for continuous variables.

## Results

### General characteristics of the study population

The study included a total of 312 cases meeting the inclusion criteria, collected over the period 2018–2024. The mean age of the subjects was 49.3 ± 12.1 years (range: 21–74 years), with a predominance of female patients (61%, *n* = 190). (Table 2).

**Table 2:** Demographic data

Variable	Value
Mean age	49.3 ± 12.1 years
Female sex	61% (*n* = 190)
Most frequent mechanism	Domestic falls (53%)
Second most frequent mechanism	Low-speed vehicle collisions (32%)
Other mechanisms	Slips (8%), sports (4%), torsion/flexion (3%)
Advanced imaging used	MRI 71%, CT 29%
Mean TTD	21.7 ± 9.8 days
Litigation	129 (41.3%), 74 included explicit allegations of medical malpractice (23.7%)

*TTD* Time to diagnosis, *MRI* Magnetic resonance imaging, *CT* Computerized tomography

**Fig. 1:**
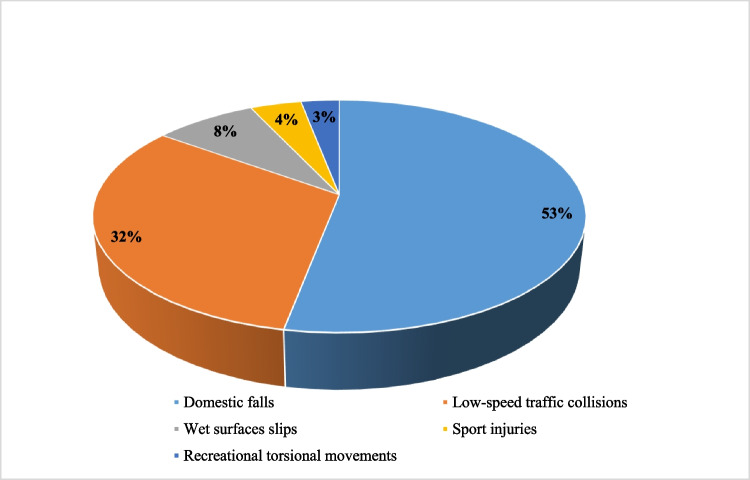
Traumatic mechanism. Pie chart showing the distribution of different traumatic mechanisms in the analyzed cases

The most frequent traumatic mechanisms were accidental domestic falls (53%, *n* = 165), followed by low-speed road traffic collisions (32%, *n* = 100). Other mechanisms included slips on wet surfaces (8%), low-impact sports injuries (4%), and indirect trauma caused by recreational sudden torsional or flexion movements (3%). (Fig. 1).

In 78% of cases, the first trauma was considered not clinically significant; consequently, no further instrumental investigations beyond standard radiography were indicated, nor was additional clinical follow-up in the discharge documentation. However, in all included cases, persistent pain, unfavorable clinical progression, and the need for medicolegal assessment of sequelae, either for the return to work or for insurance purposes, led to advanced imaging, which occult fracture diagnosis.

### Localization of occult fractures

**Fig. 2:**
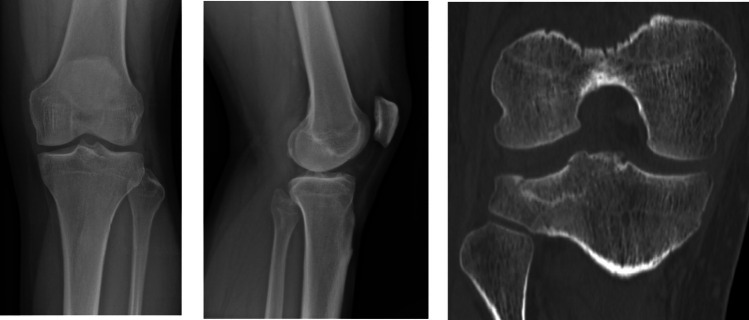
Left knee: tibial plateau occult fracture not detected on X-ray (left and middle images) and later identified on CT (right image)

The anatomical sites most frequently affected by occult fractures were distributed as follows: Sacrum (mainly alar and body regions) 29.5% (*n* = 92), ilio-ischial branch (posterior pelvic ring) 18.9% (*n* = 59), carpal scaphoid (mainly proximal portion) 16.0% (*n* = 50), lumbar transverse processes (L3–L5) 13.1% (*n* = 41), vertebral pars interarticularis (with or without lysis) 5.5% (*n* = 17), in the majority of cases involving lumbar vertebrae (3%, *n* = 10). Other less frequent sites included ribs (3.8%, *n* = 12), talus (2.9%, *n* = 9), and thoracolumbar vertebral bodies (1.6%, *n* = 5). Other sites account for 8.7% (*n* = 27) and include radius and ulna bones, navicular bone, tibial malleoli, femur, tibia, phalanges of the hands and feet, and other bones of the foot (Fig. 2).

Overall, the vertebral location accounts for 20.2% (*n* = 63), the axial location, including sacral, vertebral, and pelvic bones fractures accounts for 68.5% (*n* = 214) of the cases (underlined in Table 3), while the four most frequent sites together account for 77.5% (*n* = 242).

**Table 3:** Anatomical sites analyzed

Anatomical site	n	%	Position
Sacrum	92	29.5%	**1°**
Ilio-ischial branch (posterior pelvic ring)	59	18.9%	**2°**
Carpal scaphoid	50	16.0%	**3°**
Lumbar transverse processes	41	13.1%	**4°**
Vertebral pars interarticularis	17	5.5%	
Ribs	12	3.8%	
Talus	9	2.9%	
Thoraco-lumbar vertebral bodies	5	1.6%	
Others	27	8.7%	
**Total**	**312**	**100%**	

Underlined the sacral, vertebral, and pelvic bone fractures

Among anatomical sites involved by OFs, a further clinical distinction could be made, depending on the recommended and delayed treatment highlighting the possible major disabling consequences. When dealing with OFs, therefore minor fractures, it can be stated that in certain anatomical districts the approach is almost invariably conservative, without absolute restrictions on mobility or weight-bearing, and thus with only a limited impact of a delayed diagnosis and initiation of treatment on long-term sequelae (lumbar transverse processes, ribs, sacrum, ilio-ischial branch, fractures that do not involve the articular surface in the forearm or phalanges 207/312, 66.3%). The other sites involved by OFs could benefit significant load and mobilization restrictions (as thoraco-lumbar vertebral bodies) or surgery (as carpal scaphoid, talus, tibial malleoli, fractures that involve the joint surface in the forearm or phalanges (105, 33.7%).

In 18 out of 312 cases (5.7%), the fracture was retrospectively identified on the standard radiograph performed in the acute phase, following re-evaluation during medicolegal consultation. In the remaining 94.3% of cases, the diagnosis was made only through MRI (71%) or multislice CT (29%). The mean time to diagnosis (TDD) was 21.7 ± 9.8 days.

Mean time to diagnosis was 21.7 ± 9.8 days, and in 12.5% of cases, the diagnosis was made more than 30 days after the traumatic event, following clinical worsening or required for comprehensive medicolegal assessment.

### Forensic implications

In 129 cases (41.3%), delayed recognition of the occult fracture led to a claim for compensation motivated by the lack of prompt diagnosis. Of these, 74 cases (23.7% of the total and 57.4% of 129 claimed cases) included explicit allegations of medical malpractice, directed toward emergency department physicians (85%) and outpatient specialists (15%), for clinical underestimation and failure to recommend prompt diagnostic investigations, with alleged major harm.

In descriptive terms, from a medicolegal standpoint, the main critical issues identified were as follows:

- Lack of shared diagnostic alert criteria for minor trauma with persistent pain, resulting in the absence of recommendations for clinical or imaging follow-up.
- Inadequate documentation of clinical signs and radiological decision-making in emergency department reports.
- Minimal or overly concise descriptions of trauma dynamics and/or symptoms in initial reports, which may later be interpreted in trial as superficial or negligent.

## Discussion

The findings of this study show that occult fractures following low-energy trauma represent a category with a high medicolegal impact due to the elevated risk of claims of delayed diagnosis and treatment.

The reported anatomical sites (Table 3) were consistent with areas most exposed to stress and tears in low-energy traumas. They are in fact junction sites (lumbosacral junction with the pelvic girdle), sites exposed due to fall-protection mechanisms (wrist and hands) or more stressed by distractive or contusive forces (vertebrae or talus). The predominance of vertebral and pelvic grindle involvement in our case series must be related to the traumatic mechanisms: falls from minimal heights, slips on wet surfaces, and low-speed road accidents [15, 16]. The falls from minimal heights, often landing on the buttocks, are characterized by a perpendicular or latero-lateral vector to the pelvic plane, producing a compressive effect on the pelvic girdle, while the acceleration-deceleration mechanism in road accidents acknowledge excessive stress in flexion–extension or lateral inclination of the spine. The found prevalent axial location, approximately two-thirds of cases, could limit generalization and so the significance of the association with litigation.

The traumas dynamic could also explain the atypical prevalence of adult females compared with clinical reports, a particularly intriguing finding as it opens the way to further considerations about the possible underlying reasons for delayed diagnosis. Indeed, in clinical series, adults are predominant but are typically injured by high-energy trauma related to motor vehicle accidents, occupational injuries, or major sports trauma, including falls from significant heights, and are predominantly males [17]. By contrast, pediatric and elderly patients are more frequently involved in low-impact, domestic trauma and usually immediately present to the emergency room (EMR), often in the context of other pathological factors [18]. This could give rise to a form of cognitive bias, which leads EMR clinicians to focus attention on pediatric and elderly patients in low-energy traumas, prompting in these groups of patients further follow-up, additional investigations, or immediate observation, whereas diagnostic suspicion arises less frequently in otherwise healthy middle-aged female adults who present after minor trauma and usually after some days from the trauma [19].

The type of trauma and the forensic case mix may also explain the partial difference in the anatomical sites involved compared with the clinical-oriented literature, in which the extremities are more typically affected and where greater diagnostic complexity and higher levels of specialist expertise are required [7, 20, 21].

In a minority of cases (5.7%), the fracture was retrospectively identified on standard radiographs taken during the acute phase, due to the specific attention to the anatomical region derived from forensic assessment and purpose. [3]

In such cases, liability for missed diagnosis could be attributed to the emergency radiologist when the diagnostic query, the mechanism of injury, and the anatomical site requiring careful evaluation were clearly specified in the clinical records. It is still true, however, that diagnosis is often more challenging than the standard of care ordinarily expected of an average practitioner under the same conditions, whether due to the anatomical characteristics of the region involved, the emergency setting, or the intrinsic limitations of the imaging modality itself. In fact, when retrospectively reviewed in a forensic or insurance context, minimal signs may be found with more ease and initially interpreted as a diagnostic error, while the subtlety of the condition or the lack of information on the injury *ex ante* make the diagnosis inherently complicate [3]. In our case series, no radiologists were engaged in malpractice claim, as the patient challenged the conduct of the treating physician, alleging an underestimation of symptoms and clinical signs.

The explicit mention by the radiologists of the limitations of the imaging modality or of the vagueness in the diagnostic query in some imaging reports may help the emergency physician in recommending further investigations or indicating right clinical and therapeutic precautions or asking collegiate reading (Table 4).

**Table 4:** Key recommendations for low-energy traumas management in emergency settings

Emergency physician	Emergency radiologist	Reasoning and practical applications
Collect, take into account and trace into records, apart from clinical examination, trauma dynamics, and specify them into diagnostic imaging motivation	Refer to diagnostic imaging question, suggesting to clinicians further second level investigation in difficult-to-diagnose and more sensitive districts (hands and feet)	Trauma dynamics could allow focusing on specific anatomical sites and joints Highlight and prove high standard of care
Clearly indicate and trace into records to refer to a physician in case of persistence or worsening of signs and symptoms	Specify “with the limitation of urgency examination” or “with the limits of RX methods”	
Clearly indicate and trace into records of load, sport and weight-bearing restrictions when suffering pain		Some adult subjects perform quite daily heavy work and sport activities
Ask for second or multidisciplinary or experts (e.g. pediatric radiologist) reading of the earlier imaging in case of persistence or worsening of signs and symptoms	Describe for clinicians, when possible, surrounding tissue involvement or doubtful signs, or irregularities	Highly recommended in second evaluation, as more possible than in emergency settings
Disclose to patient the limits of emergency imaging and management, to an aware and earlier reference to specialist evaluation in case of not healing		For instance, in the informative disclosure for undergoing radiology into EMR

Conversely, the data are significant as they illustrate how, in most cases, conventional X-ray imaging fails to detect minimal fracture lines, sometimes without a complete disruption of the bone structure and with minimal involvement of surrounding tissues (edema and hematoma). This makes detection extremely complicate or needs additional ultra-specialized expertise, different to the scope and grade of sensitivity of the emergency context. This frequency is consistent with earlier reports in the literature [22, 23].

Within the inherent limits of imaging detectability in emergency settings, the radiologist’s liability in cases of occult fractures is generally considered to be absent, provided that the examination has been performed following current guidelines and that the radiologist has acted in compliance with the accepted standard of care.

The apparent mildness of the first trauma often leads to clinical underestimation, triggering a cascade that could result in delayed diagnosis and, ultimately, medicolegal disputes ground on symptoms of persistence and disability.

Unlike major trauma, which typically follows a standardized and linear diagnostic pathway, minor trauma and related fractures present challenges in terms of clinical suspicion, consistency of the patient’s history, and appropriateness of the initial diagnostic approach, particularly in the fast-paced environment of emergency departments. On the contrary, when compared to a second assessment in a planned, non-emergency and specialized setting, the standard of care expected is necessarily higher.

This is the case of over 94% of the analyzed cases, when conventional imaging proved insufficient, calling treating physicians for adequate post-discharge indications and follow-up recommendations. This underscores the concept of delayed diagnosis, where a fracture is identified later despite being reasonably suspectable based on clinical and contextual findings. Persistent pain, although subjective, is a key indicator at follow-ups for the early recognition of occult fractures and for adjusting the diagnostic and rehabilitation pathway.

It therefore becomes clear once again that the involvement of the radiologist is unlikely, as also seen in our case series. The radiologist’s assessment in emergency departments is typically guided by the diagnostic query and constrained by the inherent limitations of the imaging modality, particularly in certain anatomical regions. Rather, the overall clinical reconstruction of the case, with atypical symptoms at the initial evaluation or the persistence of unusual signs and symptoms during follow-up, represent the red flags that should raise clinical suspicion. Accordingly, medico-legal claims tend to be directed toward the clinicians responsible for patient management.

Thus, an additional issue becomes even more plain: the anatomical site of OFs and its influence on the healing trajectory, particularly in determining the need for prompt surgical or, in conservatively managed cases, the proper weight-bearing and functional limitations. In our case series, only one third of OFs (33.7%) were in more sensitive sites. However, even mild pain or sensory symptoms, such as those at the pelvic level, may be markedly disabling and frustrating. From a causal perspective, delayed management cannot be excluded as a contributing factor, even if non-surgical but conservative management was required, thus constituting the very origin of the dispute.

For the identification and management of OFs through further second level instrumental investigations, proper follow-up is essential, as pain represents both the symptom that raises diagnostic suspicion and, if inadequately managed, a major source of patient dissatisfaction. This dissatisfaction may ultimately lead to litigation. It must be clearly acknowledged that, in many cases, further second-level imaging is driven by insurance or occupational reasons, leading to the diagnosis of fractures that would likely have resolved spontaneously without care adaptation, see the minor ones involving vertebral transverse processes [24]. It is within this “grey zone” that much of the professional liability arises, not from the technical failure to recognize a fracture on imaging, but from the clinical failure to raise a diagnostic suspicion, to appreciate persistent clinical signs, or to maintain a cautious clinical-rehabilitative course aimed at full recovery. Clinical consequences arising from a missed diagnosis or delayed management of an OF not detectable at plain RX cannot reasonably be attributed to the emergency radiologist. Responsibility instead rests with the physician who must integrate negative or inconclusive imaging findings with the overall clinical assessment and determine the proper diagnostic or therapeutic pathway, including the need for additional imaging or closer follow-up. Malpractice in these phases usually burdens emergency physicians and traumatologists and later, the specialized professionals engaged, mainly orthopedics and physiatrists, or the family doctor.

Therefore, the issue is not solely technical or radiological but also pertains to the level of clinical-diagnostic responsibility. Specifically, which elements can be considered sufficient to conclude the diagnostic process. This underscores the urgency for a more systematic and shared approach that moves beyond the reductive concept of the “absence of radiographic evidence” and instead values the “evidence of reasoned clinical suspicion”, necessarily grounded on clinical reasoning [25].

The management of occult fractures, in fact, calls upon the physician’s responsibility not only to see but to recognize the potential presence of a lesion based on the clinical reasoning expected from an average professional, as in cases of refractory pain, compatible trauma dynamics, high-risk locations, atypical symptoms, and lack of spontaneous improvement. In fact, the failure to recommend adequate follow-up or further investigations in at-risk cases (e.g., athletes, traffic accidents, domestic falls with unclear dynamics, hands and feet involvement) may constitute a motivation for dissatisfaction sensation in users, and for claiming professional liability [26].

From this perspective, the time to diagnosis is not solely a chronological variable, despite the limitations inherent in a single-professional, geographically restricted case series and patient-dependent factors, but rather as an indicator of the ability to detect clinically relevant warning signs at an early stage. The analysis of the forensic process of this case series showed that a prolonged time to diagnosis represents a reason for litigation. This can be explained by the fact that a delayed detection of OF is associated with a less careful clinical and rehabilitative management, even to surgical delay, with possible more severe negative consequences and limitations in terms of functionality, quality of life, and work. Furthermore, it generates greater frustration about recovery expectations and a sense of misdiagnosis, which leads to the pursuit of a judicial solution.

However, occult fractures may appear and be perceived by patient as professional negligence when associated with symptoms and disabilities, particularly in more vulnerable individuals or those with specific needs, where there has been inadequate clinical and informative management. Brief information notes on the limitations of radiological examinations in the emergency department, included in the specific consent forms, is certainly useful; however, it indirectly highlights the lack of caution of healthcare professionals who, when symptoms persist, do not adequately assess those limitations. The selection bias of this forensic cases series is evident, but it highlights the fact that the physician that has comprehensively addressed the persistent symptoms or discomfort or post-trauma functional dissatisfaction was a forensic one, which reflects the absence of clinical care and/or further instrumental investigations along the post-trauma phase. This finding is consistent with established medicolegal literature, which recognizes diagnostic delay as one of the strongest predictors of legal conflict, regardless of the actual severity of the biological injury [27].

A further challenge in defining liability for malpractice in missed or delayed diagnosis of fractures following minor trauma arises from the timing and manner of the first medical contact. In most cases, especially those involving domestic injuries, territorial emergency services are not activated, and patients reach healthcare facilities independently, often hours or days after the event and sometimes after self-medicating with analgesics or anti-inflammatories. This data is not available in our cohort, as it is frequently not reported by the patient nor asked by clinicians. Patients involved in those traumatic mechanisms typically choose hospitals based on proximity rather than clinical complexity, expecting shorter waiting times, and therefore avoiding higher-level emergency departments. This scenario is significant because many cases are first managed in Primary Care Emergency Units or Community Hospitals, where healthcare facilities requirements ensure only traditional radiology services with remote or on-call reporting support, excluding MRI and CT, answering hospital organization [28, 29]. Furthermore, radiological interpretation may vary based on specialist availability, as far as other specialized physicians required case-by-case. The same orthopedic consultations are often secondary and at the patient’s discretion, following initial evaluation by emergency or internal medicine physicians [30]. Nonetheless, essential medical elements, such as comprehensive history (including conditions like osteoporosis or immediate analgesic use), clinical examination findings, reported symptoms, and correlation with trauma mechanism, context and timing, remain crucial and must be carefully documented to ensure both clinical accuracy and medicolegal reliability [31].

A recurring critical issue appearing from the analysis of the cases was slight medicolegal awareness. In many cases, healthcare professionals did not fully appreciate the legal and evidentiary relevance of their decisions, especially in terms of clinical records completeness, procedure adherence, and follow-up tracing. Commonly identified deficiencies included the failure to collect trauma dynamics, the decision to discharge patients based on an unclear clinical picture, and the lack of recommendations for clinical or instrumental follow-up in cases of symptom persistence or worsening.

These factors were frequent grounds for legal disputes and often made it difficult for clinicians to prove adherence to proper standards of care, after the patient alleged malpractice (lack of or delayed clinico-surgical take in charge) and the harm according to a reasonable and valid causal link. Even in minor trauma, temporary disability and functional limitation have legal and economic repercussions that can easily lead to complaints and compensation claims [32].

Some key recommendations for addressing possible medicolegal issues in low-energy traumas were synthetically provided in Table 4.

Some limitations must be disclosed. The more relevant is the selection bias, which results in a higher prevalence of occult fractures in low-energy traumas than a simple clinical case series, as well as a greater prevalence of critical issues from which malpractice claims arose. The average time to first healthcare contact is not defined, which may indicate a delayed presentation to the hospital by the patient, nor is any additional harm clearly associated with an increase in TTD defined and the final complaint outcome, depending on judicial timing and manners. Finally, the case series derives from a single geographical area but has the homogeneity of evaluation by a single expert assessor.

Considering the insurance context, it would be useful to retrospectively assess fractures that were identified in a clinical setting during second level imaging analyses, such as MRI, to verify the presence of the fracture at traditional radiology, as well as the diagnostic and therapeutic indications, specifically looking for diagnostic query, trauma description, anatomical site involved.

## Conclusions

Low energy trauma is a possible cause of missed fracture identification in emergency care settings, with significant medicolegal implications, as these injuries are often diagnosed only upon re-evaluation, leading to delayed treatment and motivating malpractice claims.

They show peculiar epidemiological patterns, anatomical locations, and trauma dynamics, which require further investigation with broader representativeness. Diagnostic delay typically alters both therapeutic and prognostic pathways, particularly when a surgical approach is required, paving the way for more severe and disabling consequences than would be expected with prompt management. These features, together with insufficient documentation of post discharge instructions, functional rest, or follow-up, and the fact that these cases often require forensic expertise for insurance purposes, underline an elevated risk of litigation.Therefore, occult fractures in low-energy trauma represent a transversal phenomenon across clinical practice, radiology, and forensic medicine, calling for comprehensive multidisciplinary attention. Implementing standardized protocols for the management and documentation of mild and moderate trauma cases within EMR could improve patient care, strengthen the quality of diagnostic documentation, and reduce litigation rates.

## Data Availability

All data generated or analysed during this study are included in this published article.

## Abbreviations

OF: Occult fracture
TTD: Time to diagnosis
MRI: Magnetic resonance imaging
CT: Computerized tomography
EMR: Emergency room
DDI: Delayed diagnosed injuries
